# PRISMA-based review of *Pseudomonas* spp. in microbial heavy-metal bioremediation: mechanisms and taxonomy

**DOI:** 10.3389/fmicb.2026.1760258

**Published:** 2026-02-27

**Authors:** Alessandro De Santis, Maria Rosaria Corbo, Angela Racioppo, Antonio Bevilacqua, Milena Sinigaglia

**Affiliations:** Department of Agriculture, Food, Natural Resources and Engineering, University of Foggia, Foggia, Italy

**Keywords:** bacterial bioremediation, efflux and redox mechanisms, heavy metal detoxification, *Pseudomonas* spp., systematic review

## Abstract

**Introduction:**

Bioremediation may be sustainable strategy for mitigating heavy-metal contamination in soils and related environmental matrices.

**Methods:**

This paper, based on PRISMA 2020 guidelines analyzes 41 experimental studies published between 2022 and 2025, selected from Scopus, Web of Science, and PubMed, with the aim of comparing bacterial taxa, detoxification mechanisms, and remediation performance.

**Results and discussion:**

*Bacillus* and *Pseudomonas* were the most frequently investigated genera, with *Bacillus* spp. achieving the highest removal efficiencies (>80%–90%) through biosorption and biomineralization mechanisms. On the other hand, *Pseudomonas* spp. showed a high functional and genetic versatility, combining efflux systems, enzymatic redox transformations, EPS production, and biofilm-mediated resilience, particularly in multi-metal and fluctuating environmental conditions. Median removal efficiencies was at least 70% in controlled laboratory assays, while it was 10%–25% in soil. Despite methodological heterogeneity, the evidence identifies *Pseudomonas* as a mechanistic benchmark genus rather than a universally superior biosorbent, owing to its extensive genomic characterization and broad detoxification repertoire. The review highlights the need for standardized experimental protocols, integration of multi-omics approaches, and field-scale validation to improve data comparability and to facilitate the translation of microbial bioremediation from laboratory studies to sustainable environmental applications.

## 1 Introduction

Heavy-metal contamination remains one of the most persistent environmental threats worldwide, arising primarily from industrial effluents, mining activities, intensive agriculture, and urban waste discharge. Metals such as cadmium (Cd), lead (Pb), chromium (Cr), nickel (Ni), copper (Cu), zinc (Zn), mercury (Hg), and arsenic (As) are non-degradable and can accumulate in soils and aquatic systems over time, entering the food chain and posing severe ecological and health risks (Angon et al., 2024). Their persistence leads to long-term toxicity, oxidative stress, and metabolic disruption in plants, animals, and humans. Recent assessments indicate that approximately 14%–17% of global agricultural soils are affected by excessive heavy-metal concentrations, potentially exposing more than one billion people to hazardous levels (Hou et al., 2025).

To address these concerns, multiple remediation technologies have been implemented, including chemical precipitation, ion exchange, adsorption, membrane filtration, and electrochemical treatment (Shofia et al., 2025). Although these physicochemical approaches can achieve high removal efficiencies under controlled conditions, they are often limited by high costs, secondary waste generation, and reduced effectiveness in complex or heterogeneous matrices (Ilyas et al., 2024; Oladimeji et al., 2024). Moreover, they do not restore ecological balance and can disturb soil structure or microbial functionality. Consequently, sustainable alternatives based on biological processes have gained attention as self-regulating and environmentally benign solutions.

Bioremediation, that is the use of living organisms to detoxify or immobilize contaminants, has become a central component of modern environmental biotechnology. Biological systems exploit the inherent ability of microorganisms and plants to transform pollutants into less toxic or less mobile forms. Within this framework, microbial bioremediation, using bacteria and Archaea, offers significant advantages due to its adaptability, scalability, and ability to function *in situ* under variable physicochemical conditions (Ruan et al., 2024). Recent reviews emphasize that microorganisms represent a versatile platform for heavy-metal removal in both aquatic and terrestrial environments, outperforming many traditional techniques in sustainability and cost-efficiency (Firincã et al., 2025).

Microbial communities naturally interact with metals through a combination of physicochemical and metabolic processes that promote detoxification and stabilization. Although the specific mechanisms, such as surface binding, accumulation, and transformation, differ among taxa, all rely on fundamental adaptive responses that reduce metal bioavailability and toxicity (Tang et al., 2024). The overall efficiency of microbial bioremediation depends on species composition, environmental parameters (pH, temperature, redox conditions), and metal speciation. Recent studies confirm that mixed microbial consortia frequently outperform single isolates, as interspecies cooperation enhances resilience, broadens substrate range, and stabilizes performance under fluctuating conditions (Liu et al., 2023).

A growing body of literature highlights the diversity of bacterial families capable of mediating heavy-metal remediation. Bacillaceae species exhibit strong tolerance to metals such as Pb, Cd, Hg, Cr, and Ni, owing to their robust cell envelopes and ability to function under nutrient-limited conditions. Rhodococcaceae and related Actinomycetes are known for their hydrophobic surfaces and oxidative metabolism that favor metal transformation. Shewanellaceae and Geobacteraceae can reduce metal ions through extracellular electron transport, contributing to immobilization in sediments (Tang et al., 2024). In addition, the role of extracellular polymeric substances (EPS) has been increasingly recognized as a structural and functional determinant in microbial systems, enhancing metal sequestration and biofilm stability under stress (Pan et al., 2023).

Within this microbial landscape, the family Pseudomonadaceae, particularly the genus *Pseudomonas*, stands out as a cornerstone of bacterial bioremediation. *Pseudomonas* species are cosmopolitan, fast-growing, and metabolically versatile organisms that thrive in diverse habitats, including soils, sediments, industrial effluents, and wastewater treatment plants (Ajmi et al., 2025). They are frequently isolated from multi-metal-contaminated sites and are recognized for their exceptional resistance and detoxification capacity. Recent studies have shown that *Pseudomonas aeruginosa*, *Pseudomonas putida*, and *Pseudomonas fluorescens* can tolerate and remove various metals, including Cu, Zn, Pb, and Cr, while maintaining high growth rates under stress (Zada et al., 2025). Their ecological success is attributed to their capacity to form biofilms, secrete siderophores, and regulate gene networks associated with metal homeostasis and oxidative-stress management (Dey et al., 2025). These features not only confer resilience but also make *Pseudomonas* spp. ideal candidates for bioaugmentation and biosensing applications.

Despite the substantial progress achieved in the last decade, the current scientific landscape remains methodologically fragmented. Many studies are case-specific, focusing on individual isolates, single metals, or specific experimental conditions, with limited comparability across datasets (Ghorbani and Amirahmadi, 2025). In addition, critical operational parameters such as pH, temperature, exposure time, and analytical techniques are often inconsistently reported. This heterogeneity hampers quantitative synthesis and prevents the identification of generalizable patterns linking bacterial taxa to remediation performance. Addressing these limitations requires a rigorous and transparent framework that can harmonize experimental evidence and facilitate reproducible data interpretation.

This systematic review integrates quantitative outcomes (removal efficiency and sorption capacity), environmental variables (pH, temperature, matrix), and taxonomic distribution across bacterial families, while maintaining a focused interpretative perspective on the genus *Pseudomonas*. In addition to the experimental synthesis, a bibliometric analysis was performed using VOSviewer, which enabled visualization of the most frequent keywords, co-authorship networks, and thematic clusters emerging from the recent literature. This complementary approach provided an overview of research trends, identifying the dominant conceptual domains and highlighting the growing scientific interest toward *Pseudomonas*-mediated heavy-metal remediation. By synthesizing recent empirical evidence, this work aims to identify comparative trends among bacterial groups, outline optimal conditions for heavy-metal removal, and highlight knowledge gaps that can guide future mechanistic and applied research. Ultimately, the review seeks to establish a data-driven foundation for designing scalable, efficient, and ecologically sustainable microbial bioremediation strategies, positioning *Pseudomonas* as a benchmark genus within the broader bacterial community.

## 2 Materials and methods

### 2.1 Bibliometric analysis

A bibliometric evaluation was performed using VOSviewer (v. 1.6.20) based on extracted keywords. The dataset was derived from Scopus metadata exported as a Tables–delimited text file and processed to map co-occurrence relationships among author and indexed keywords.

The dataset was obtained from Scopus using the following query string:

TITLE-ABS-KEY (“microbiota” OR “bacteria” OR “microorganism*”) AND TITLE-ABS-KEY (“heavy metals” OR cadmium OR lead OR chromium OR nickel OR mercury OR arsenic OR copper OR zinc) AND PUBYEAR > 2,000 for a first search, and then restricted to 2022–2025.

A threshold of minimum 50 occurrences per term was applied to ensure statistical robustness. The software generated a network visualization where node size corresponds to keyword frequency, link thickness to co-occurrence strength, and color coding to cluster affiliation.

### 2.2 Review protocol and framework

This systematic review was conducted according to the PRISMA 2020 guidelines (Page et al., 2021) to ensure transparency, reproducibility, and methodological rigor. The protocol was defined *a priori* and included a description of the research question, databases, search strategy, inclusion/exclusion criteria, data-extraction procedures, and synthesis methods; the checklist is available in the Supplementary Table 1. The primary aim was to identify, analyze, and compare recent studies (2022–2025) addressing bacterial bioremediation of toxic heavy metals across environmental matrices. The central research questions were:

- Which are the bacterial genera exhibiting the highest performance in removing or transforming heavy metals?
- What are the environmental and operational conditions (pH, temperature, matrix) optimizing removal efficiency?
- How do study design and mechanistic characterization affect data comparability?

### 2.3 Information sources and search strategy

Three major databases, Scopus, Web of Science (WoS), and PubMed, were systematically queried to identify peer-reviewed research articles and reviews published between January 2022 and June 2025. Searches were limited to English-language publications and subject categories related to environmental science, biotechnology, and microbiology. Equivalent Boolean search strings were adapted for each database to ensure semantic and structural consistency. Minor syntactic differences were due to database-specific query syntax.

Before defining the final search strings, several preliminary and more permissive query combinations were tested across the three databases. These exploratory strings included broader taxonomic and functional terms (e.g., microorganism, biodegradation, pollutant removal, metalloid detoxification) to evaluate retrieval coverage and background noise. After iterative refinement, the most specific and reproducible queries were selected and applied in the final systematic search.

The final search strings, which generated the dataset used in this review (6*^th^* text string), is reported in Supplementary Table 2. The last search was performed in September 2025. Reference lists of retrieved papers were manually screened to capture additional relevant studies not indexed under the same keywords.

### 2.4 Eligibility criteria study selection

Studies were considered eligible for inclusion if they met all the following criteria.

First, only original experimental research published in peer-reviewed international journals from January 2022 onward and written in English was included. Furthermore, the following relevant parameter were used as including markers:

- Studies focused on microbial bioremediation of heavy metals in environmentally relevant matrices (soil, water, sediment, wastewater, or sludge).
- Focus on microorganisms served as the primary remediation agents, including both single isolates, and microbial consortia.
- Studies providing quantitative evidence of metal removal, tolerance, or biosorption, reporting at least one measurable parameter like removal percentage, adsorption capacity, minimum inhibitory concentration (MIC), or initial and/or final metal concentration.
- Studies including mechanistic or molecular characterization, demonstrating the biochemical, structural, or genetic basis of metal removal.
- Reported mechanisms like extracellular polymeric substances (EPS) production, biofilm formation, redox transformation, precipitation or biomineralization, expression of resistance genes.
- Mechanistic evidence supported by instrumental analyses (FTIR, SEM/EDS, XRD, TEM) or omics-level approaches (WGS, transcriptomics, metagenomics).
- Metagenomic or microbiome studies were included only if they provided quantitative data on genes or pathways directly involved in heavy-metal detoxification or resistance.

Studies were excluded if they met one or more of the following conditions:

- Plant-centered phytoremediation – experiments in which the core remediation mechanism was plant-based, even when assisted by plant growth-promoting rhizobacteria (PGPR).
- Review or theoretical papers – including reviews, mini-reviews, opinion articles, or purely conceptual frameworks without new experimental data.
- Non-metal pollutants only – investigations limited to microplastics, antibiotics, or organic contaminants not coupled with heavy-metal bioremediation.
- Lack of quantitative data – studies reporting only qualitative or descriptive outcomes without explicit numerical metrics of removal or tolerance.
- Modeling or simulation only, *in silico*, docking, or predictive models without experimental validation.
- Duplicate or inaccessible full text, articles not retrievable or overlapping with already included datasets.

A detailed summary of all inclusion and exclusion criteria applied in this review is provided in Supplementary Table 3.

### 2.5 Study selection

All records retrieved from Scopus, Web of Science, and PubMed were exported to Microsoft Excel for centralized management. Duplicate entries were identified and removed through automated matching of DOI, title, and first author fields, followed by manual verification.

Title and abstract screening was independently performed by two reviewers according to pre-established eligibility criteria. Each record was classified as Include, Maybe, or Exclude. Disagreements were resolved through discussion until a consensus was reached. Studies marked as Maybe were retained for full-text evaluation. The entire screening workflow and decision log were managed in a structured Excel matrix to ensure transparency and reproducibility.

Inclusion criteria required that (i) bioremediation represented the primary focus of the study, (ii) the biological system consisted of bacterial isolates or microbial consortia, (iii) at least one of the target metals was Cd, Pb, Cr, Ni, Cu, Zn, Hg, or As, (iv) quantitative removal data were reported (e.g., initial and residual concentrations, percentage removal, adsorption capacity, or kinetic parameters), (v) robust analytical methods were applied (ICP-MS, AAS, ICP-OES, SEM-EDS, FTIR, or XRD), and (vi) taxonomic identification was provided at least to the 16S rRNA gene level.

Studies were excluded if they focused exclusively on phytoremediation or non-metal pollutants (e.g., dyes, hydrocarbons, pesticides), employed aquatic or wastewater systems without soil relevance, lacked quantitative outcomes, failed to identify microorganisms, or represented reviews, commentaries, or duplicated datasets.

All records (188) retrieved from Scopus (*n* = 98), Web of Science (*n* = 86), and PubMed (*n* = 4) were imported into Microsoft Excel for centralized management and deduplication. Duplicate entries were identified through automated DOI matching and manually verified; 28 duplicates were removed, resulting in 160 unique studies eligible for screening.

**FIGURE 1:**
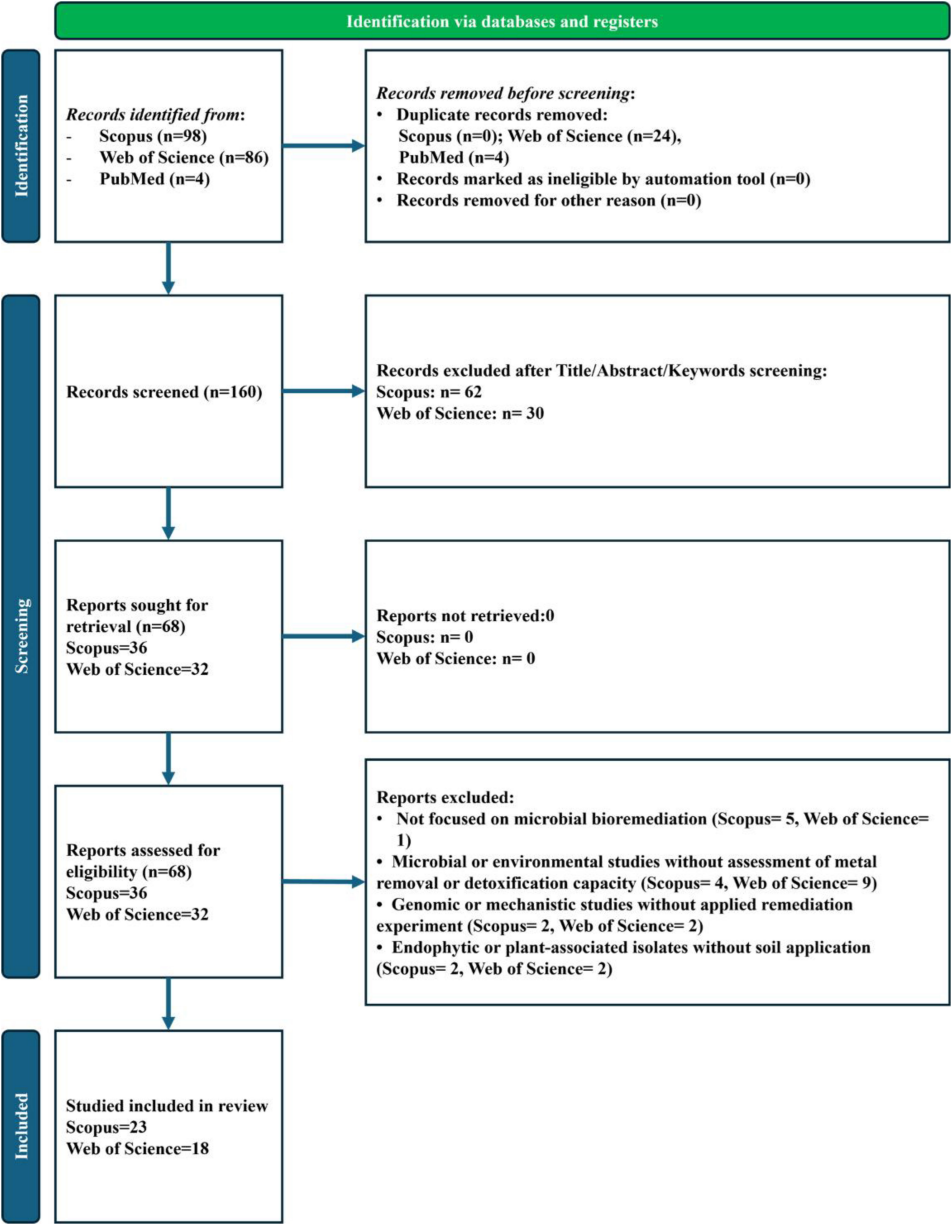
PRISMA 2020 flow diagram summarizing the identification, screening, eligibility, and selection process for the systematic review. A total of 188 records were initially identified across Scopus (*n* = 98), Web of Science (*n* = 86), and PubMed (*n* = 4). After removing 28 duplicates, 160 unique records were screened based on title, abstract, and keywords. A total of 68 full texts were assessed for eligibility, and 41 studies met all inclusion criteria and were finally included in the review in accordance with the PRISMA 2020 statement.

Title, abstract, and keyword screening was independently performed by two reviewers according to the inclusion and exclusion criteria reported in Supplementary Table 3. After this phase, 68 studies were considered potentially relevant and retrieved for full-text assessment. Following detailed evaluation, 41 studies met all eligibility requirements and were included in the systematic review. The main reasons for exclusion were: (i) absence of quantitative data on heavy-metal removal; (ii) focus on phytoremediation or non-metal pollutants; (iii) lack of taxonomic identification; and (iv) studies not performed on soil or soil-related matrices. The complete identification, screening, and inclusion process is summarized in Figure 1, which follows the PRISMA 2020 flow-diagram structure. The corresponding PRISMA 2020 checklist is provided in the Supplementary materials.

### 2.6 Data extraction

For each included article, a structured data-extraction form was completed to capture, when applicable:

- Bibliographic metadata: authors, year, journal, DOI;
- Taxonomic data: genus, species, strain code;
- Target metal(s) and initial concentration;
- Matrix type (soil, effluent, sludge, synthetic medium);
- Experimental conditions: pH, temperature, contact time;
- Analytical techniques (AAS, ICP-OES, ICP-MS, XRF);
- Quantitative performance metrics: removal%, q_max_, rate constants;
- Mechanistic evidence (biofilm formation, EPS, enzyme assays, redox changes).

Data were independently extracted by two reviewers and cross-checked for consistency. Discrepancies were resolved through consensus.

### 2.7 Quality assessment

The methodological quality of the included studies was evaluated using a modified Newcastle–Ottawa Scale (NOS) adapted for environmental and microbiological research. Each study was assessed on the following domains, as reported in Table 1.

**TABLE 1:** Modified Newcastle–Ottawa Scale (NOS) criteria adapted for evaluating the methodological quality of environmental microbiology studies included in the review.

Criterion	Description	Scoring
Study design	Clear experimental setup and control conditions	0–2
Taxonomic identification	Level of bacterial identification (genus/species/strain)	0–2
Quantitative validity	Analytical precision (AAS, ICP-OES/MS, replicates)	0–2
Mechanistic characterization	Evidence of mechanism (EPS, enzymes, biofilm, redox)	0–2
Reproducibility and reporting	Complete reporting of methods and statistics	0–2

A maximum score of 10 indicated high methodological quality. Studies scoring ≥7 were retained for full synthesis; those below were discussed qualitatively. To strengthen the methodological robustness of the synthesis, the quality assessment based on NOS was also analyzed according to bacterial genus and target metal. For each genus–metal combination represented by at least three studies, mean NOS scores, standard deviations, and score ranges were calculated. The results are in the Supplementary Tables 4, 5.

### 2.8 Data synthesis

A mixed-methods synthesis approach was applied:

- Quantitative aggregation of removal efficiencies by bacterial genus and metal type;
- Qualitative thematic analysis of recurring mechanisms, experimental conditions, and ecological context.

Statistical summaries (mean ± SD, range, median) and visualizations (boxplots, heatmaps) were generated in Statistica v.14 (Statsoft, Tulsa, OK).

Results were organized thematically into four domains:

1. Taxonomic distribution and frequency;
2. Metal-specific performance;
3. Matrix influence and environmental conditions;
4. Mechanistic diversity and adaptation strategies.

## 3 Results

### 3.1 Bibliometric network of keywords

**FIGURE 2:**
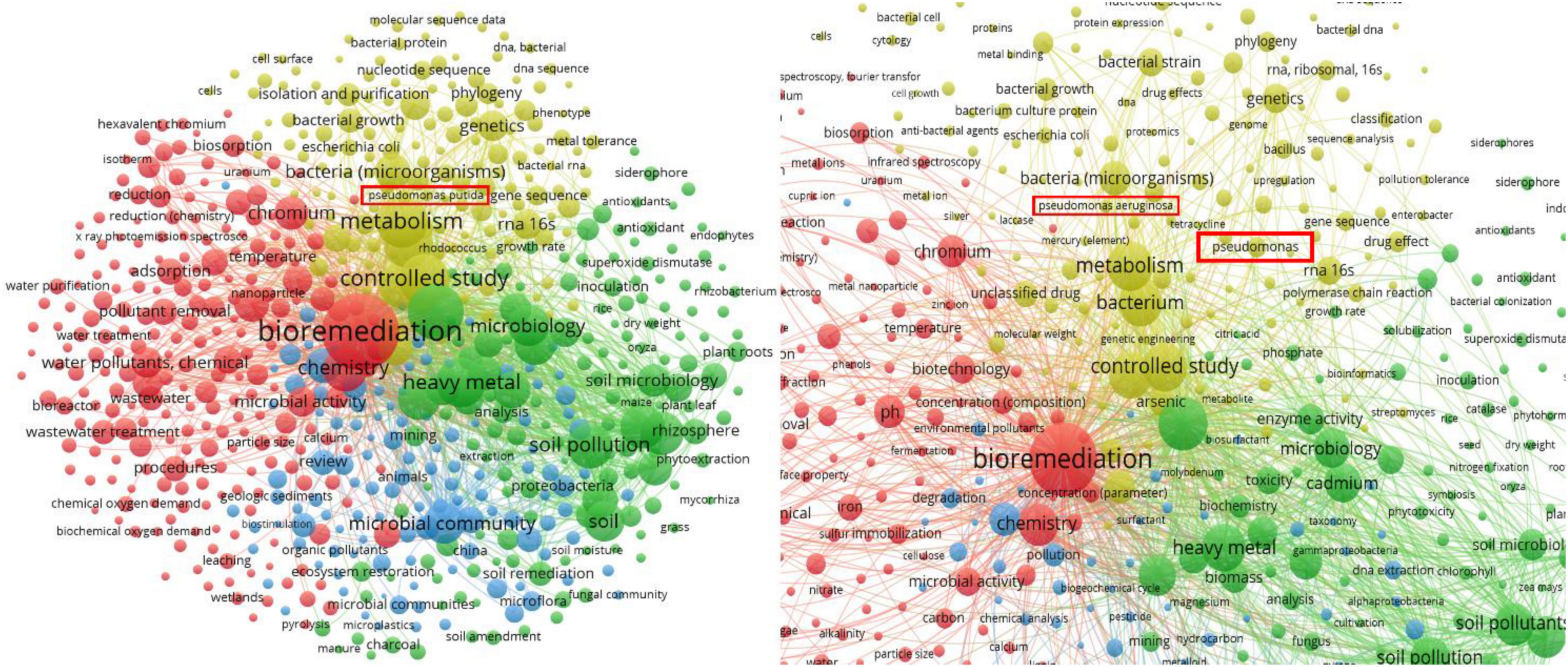
VOSviewer network of keyword co-occurrence in microbial heavy-metal bioremediation studies (2000–2025). Node size represents keyword frequency (occurrence ≥ 50) and link thickness indicates co-occurrence strength. Colors denote main thematic clusters: red (bioremediation and biosorption), green (soil pollution and microbial communities), yellow (genetics and *Pseudomonas*-related studies), and blue (environmental engineering and water/sediment remediation). Left side: whole VosViewer analysis; right side: focus on *Pseudomonas*.

The bibliometric co-occurrence analysis conducted via VOSviewer revealed a structured network of research themes within the field of microbial heavy-metal bioremediation (Figure 2). Four major clusters emerged: (1) “bioremediation/heavy metals/removal” (red), characterized by high frequencies of nodes such as “bioremediation,” “adsorption,” “chromium,” “biosorption”; (2) “soil pollution/microbial community” (green), containing keywords like “soil,” “microbial community,” “plant growth,” “soil pollutants”; (3) “genetics/metabolism/bacterial taxonomy” (yellow), including “metabolism,” “genetics,” “*Pseudomonas*,” “metal tolerance”; and (4) “environmental engineering/water/sediment” (blue), encompassing “water pollution,” “sediment,” “ecosystem restoration.” The size of nodes (frequency of keyword occurrences) and thickness of links (co-occurrence strength) indicate that the red and green clusters are the dominant thematic domains, with “bioremediation” and “soil pollution” serving as central hubs. The dense interconnections between clusters highlight that research on microbial remediation of heavy metals is inherently multidisciplinary, bridging microbial physiology, soil science, pollutant removal technologies and ecosystem restoration. Notably, keywords associated with the genus *Pseudomonas* and “metal tolerance” are centrally located within the yellow cluster, thus supporting the decision to treat *Pseudomonas* as a benchmark genus in this review. The complete list of keywords ranked by frequency and link strength is provided in Supplementary Table 6.

### 3.2 General overview and quantitative performance of the included studies

#### 3.2.1 Quality assessment

The results for quality assessment for the selected studies (41) are in the Supplementary Table 4. All selected papers had a NOS score of at least 7, with a maximum score of 9. NOS stratification as a function of bacterial genera and metals reveals some differences (Supplementary Table 5). Papers on *Pseudomonas* spp. generally exhibited higher average NOS scores, mainly for chromium and arsenic detoxification. This trend was primarily associated with more frequent inclusion of genetic, enzymatic, and spectroscopic validation of removal mechanisms. On the other hand, studies centered on *Bacillus* spp., while reporting higher removal efficiencies, showed slightly lower average NOS scores due to more limited mechanistic characterization. Focusing on the target metal, papers on Cr(VI) showed the highest overall methodological quality, reflecting the frequent use of redox-specific assays, gene-level validation, and complementary analytical techniques, while studies on cadmium and lead had a large variability in quality, particularly in soil-based systems where environmental complexity constrained experimental control.

The evaluation of studies with lower NOS scores were generally laboratory-based, reporting high removal efficiencies but with a limited mechanistic evidence. However, they did not alter genus- or metal-trends.

#### 3.2.2 Dominant genera and distribution patterns

From a taxonomic standpoint, *Bacillus* spp. dominated the dataset (*n* = 23), followed by *Pseudomonas*, *Lysinibacillus*, *Enterobacter*, and *Staphylococcus*. *Bacillus* species were the most frequently isolated from industrial or agricultural soils contaminated with Cd, Pb, or Cr, often achieving removal efficiencies above 80%. *Bacillus altitudinis* and *B. tequilensis* reached Pb removal rates close to 98% under slightly alkaline conditions (pH 7.5–8.0), whereas *B. cereus* exhibited strong Cd and Cr(VI) biosorption coupled with carbonate precipitation (Zuo et al., 2022; Kaushal and Pati, 2024; Chen et al., 2025; Wang et al., 2025). *P. aeruginosa* and *P*. *putida* displayed greater versatility across metal types, with efficient Cr(VI) to Cr(III) reduction (up to 95%) within 48–72 h under aerobic conditions (Sundarraj et al., 2023; Dahnoun et al., 2024; Satyapal et al., 2024). *Enterobacter* sp. further contributed through EPS-mediated binding and urease-induced mineralization of Cd, Pb, and As (Mondal et al., 2025). While mixed consortia combining *Bacillus* and *Pseudomonas* were utilized (Mungla et al., 2022), the highest efficiencies were often observed in synergistic microbial associations. For instance, the cooperation between *Bacillus thuringiensis* and *Citrobacter freundii* demonstrated a synergistic removal efficiency of 97.68% for Cd, significantly higher than the 66%–88% achieved by either strain alone (Cai et al., 2025). Similarly, a bifunctional community composed of *Enterobacter* and *Bacillus* genera was constructed, which not only removed 98.0% of Cd via biomineralization (inducing CdCO_3_ precipitation) but also simultaneously degraded 88.3% of the organic pollutant pyrene (Lin et al., 2022). The mechanisms underpinning these synergies were linked to the induction of precipitation *via* Microbially Induced Calcite Precipitation (MICP) (Yan et al., 2024) and the upregulation of specific metabolic pathways, such as the urea cycle, which helps buffer environmental pH (Cai et al., 2025).

#### 3.2.3 Metal targets and environmental context

Across contaminants, Cd and Pb were the most frequently studied metals, often investigated concurrently in co-contaminated agricultural soils (Zuo et al., 2022; Zhang et al., 2024; Mondal et al., 2025). The highly toxic hexavalent chromium [Cr(VI)] was another primary focus, particularly in studies involving industrial effluents (Elahi et al., 2022; Ye et al., 2024). Other metals, like Ni (Kashyap et al., 2022; Manikandan and Nair, 2023), Zn (Mtengai et al., 2022; Yasmin et al., 2022), and As (Satyapal et al., 2024; Mondal et al., 2025), were also investigated. The experimental contexts varied widely, from remediating agricultural soils, where the goal was often to reduce metal bioavailability and plant uptake (Zuo et al., 2022; Dahnoun et al., 2024), to the detoxification of industrial effluents (Elahi et al., 2022; Kashyap et al., 2022), and controlled aqueous laboratory systems designed to define removal mechanisms (Manikandan and Nair, 2023; Shaaban Emara et al., 2023).

#### 3.2.4 Analytical approaches and experimental parameters

Analytical methods were highly consistent across the studies. Metal quantification was predominantly performed using Atomic Absorption Spectroscopy (AAS) (Bhandari et al., 2024; Dahnoun et al., 2024; Kaur et al., 2024; Surabhi et al., 2025) or ICP-MS (Cai et al., 2025; Chen et al., 2025; Liang et al., 2025). To elucidate removal mechanisms, FTIR spectroscopy was widely applied, confirming that biosorption relies on the involvement of key functional groups on the cell wall—primarily carboxyl (C = O), amino (N-H), hydroxyl (O-H), and phosphate (P = O) groups—which serve as binding sites for metal cations (Yasmin et al., 2022; Yan et al., 2024; Mondal et al., 2025). These spectroscopic findings were visually corroborated by SEM-EDS, which confirmed metal adhesion, accumulation, and morphological changes on the bacterial surface (Shan et al., 2024; Yan et al., 2024; Chen et al., 2025). Furthermore, XRD analysis was used to identify the specific crystalline nature of bioprecipitated minerals, such as CdCO_3_ (Ghosh et al., 2024; Yan et al., 2024; Mondal et al., 2025).

#### 3.2.5 Genetic determinants of metal resistance

Gene-level characterization identified bacterial tolerance determinants such as the efflux gene *chrA* and the metallochaperone *copZ* (Su et al., 2022). In plant-bacteria interaction studies, inoculation also demonstrated effects on the host plant; for example, bacterium H27 significantly reduced the expression of the plant’s endogenous Cd transport genes, *HAM2* and *IRT1*, in tobacco (Gao et al., 2024). Experimental conditions were typically mesophilic, optimized around pH 6.5–8.0 (Yasmin et al., 2022) and temperatures of 28 °C–40 °C (Monga et al., 2022; Yasmin et al., 2022; Shan et al., 2024). Contact times in batch biosorption assays ranged from rapid removal within 20 h (Zuo et al., 2022) to 5–7 days required for complete Cr(VI) reduction or maximum accumulation (Elahi et al., 2022; Su et al., 2022). Soil pot experiments assessing plant uptake necessarily lasted much longer, (e.g., 40 days) (Zhang et al., 2023).

#### 3.2.6 Quantitative removal efficiencies

Quantitatively, the highest removal efficiencies were consistently recorded in systems targeting Cd, Pb, and Cr(VI) (Elahi et al., 2022; Lin et al., 2022; Manikandan and Nair, 2023; Shaaban Emara et al., 2023; Bhandari et al., 2024; Kaushal and Pati, 2024; Mondal et al., 2025). The *Bacillus* genus was a dominant and highly effective performer: *B. altitudinis* showed 96% Pb bioaccumulation efficiency (Kaushal and Pati, 2024), *B. amyloliquefaciens* achieved 99.8% Pb bioremediation (Shaaban Emara et al., 2023), and *B. cereus* removed 99% of Cr(VI) from tannery effluents (Elahi et al., 2022). Other genera also showed high potential; *Lysinibacillus cavernae* demonstrated robust Cr(VI) reduction at 76.21% under optimized conditions (Shan et al., 2024). *Pseudomonas* strains showed varied but high potential, with *P. stutzeri* immobilized on rice husk biochar removing 95% of Cd and 92% of Ni (Manikandan and Nair, 2023), although other strains showed more moderate efficiencies around 60%–70% (Mtengai et al., 2022). Finally, *Enterobacter* sp. (ACP-1) achieved exceptional removal rates of 98% (Pb) and 97% (Cd) from mixed-metal solutions (Mondal et al., 2025), underscoring the high efficiencies found in non-*Bacillus* genera as well.

### 3.3 Mechanistic insights

**FIGURE 3:**
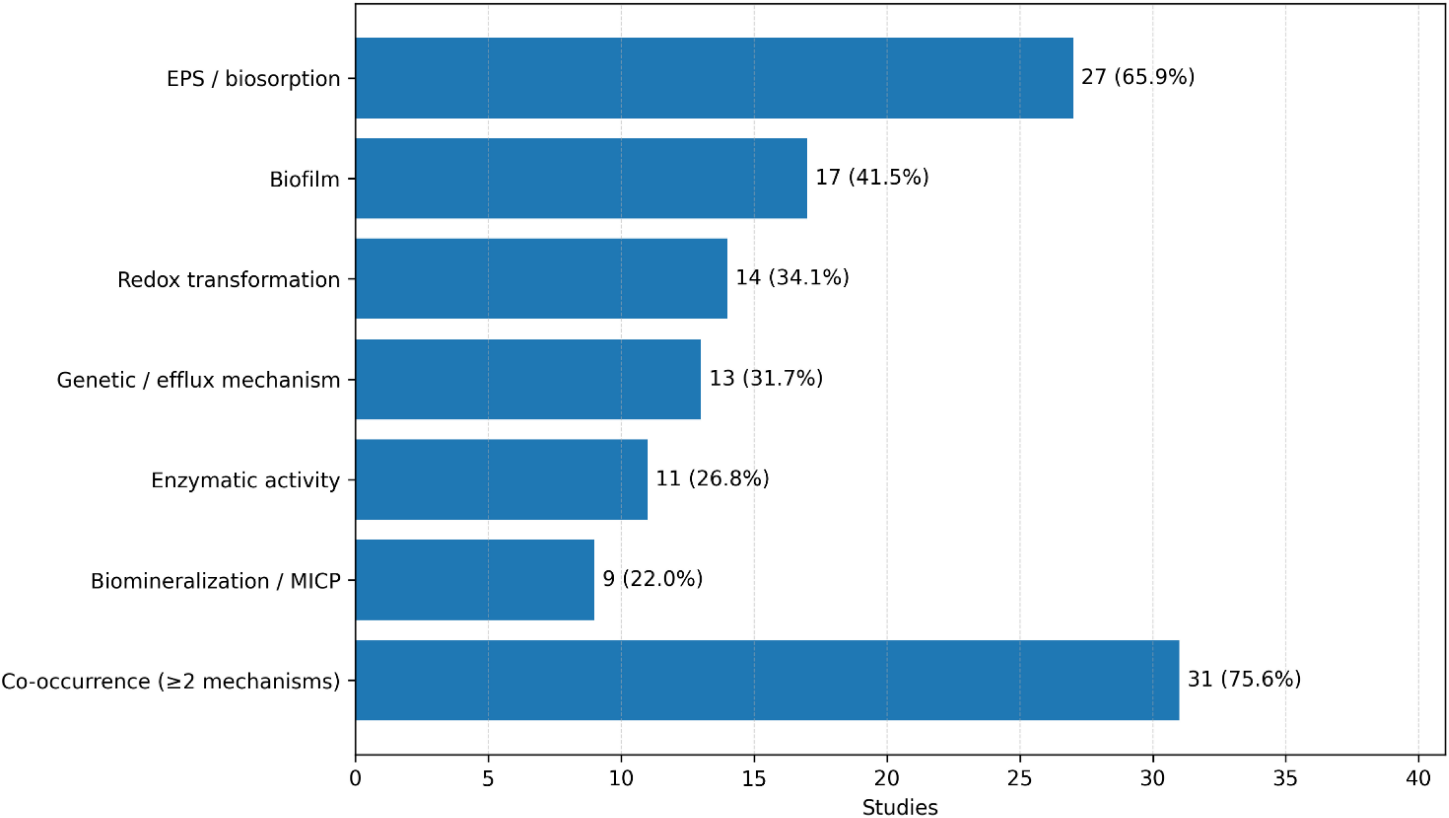
Summary of the dominant heavy-metal detoxification mechanisms identified across the dataset (*n* = 41 studies), and their frequency of occurrence. For each bar, the first number represents the number of studies, while in the brackets there is occurrence (relative frequency).

The 41 studies reveal a consistent convergence toward six dominant functional pathways (Figure 3) governing bacterial heavy-metal detoxification: EPS-mediated biosorption, biofilm-driven community resilience, enzymatic and redox transformations, biomineralization and MICP, and genetic/efflux-based regulation. These mechanisms frequently co-occur within the same strain or consortium, underscoring the multi-layered adaptability of environmental bacteria under metal stress (Manikandan and Nair, 2023; Yan et al., 2024).

Extracellular polymeric substances-mediated biosorption represented the most widespread process, documented in 65.9% of the studies. EPS secreted by *Bacillus*, *Enterobacter*, and other species provided abundant binding sites capable of complexing Pb^2+^, Cd^2+^, and Ni^2+^ ions (Chen et al., 2025; Mondal et al., 2025; Wang et al., 2025). FTIR and SEM-EDS analyses consistently identified these functional groups (such as carboxyl, amino, and phosphate groups) and confirmed their participation in surface complexation (Yasmin et al., 2022; Yan et al., 2024; Mitra et al., 2025). The strong correlation between EPS density and removal efficiency was associated with the high performance of *Bacillus* in direct biosorption assays (with efficiencies up to 99.8% for Pb) (Shaaban Emara et al., 2023; Bhandari et al., 2024).

Biofilm formation and community-level resilience appeared in roughly 41.5% of the dataset, mainly among *Pseudomonas*, *Bacillus*, and mixed consortia, specifically noted in studies on fungal-bacterial and *B. subtilis* biofilms (Henagamage et al., 2022; Ghosh et al., 2024). In rhizospheric systems, microbial consortia stabilized metal ions at the root interface, reducing plant uptake by 50%–80% (Zhang et al., 2023; Gao et al., 2024). The co-occurrence of EPS production and biofilm formation was frequently reported in studies describing the persistence of microbial populations under chronic metal exposure.

Genetic and efflux-based mechanisms (31.7%) were confirmed through the detection of specific resistance genes (Table 2). The most clearly identified mechanisms in the dataset relate to chromium (Cr) and arsenic (As). For instance, in *P. putida*, the chromate reductase (ChrR) gene was explicitly isolated and transferred to *E. coli* to confirm its responsibility for Cr(VI) reduction (Sundarraj et al., 2023). Further genomic analysis on a *Pseudomonas/Rhodococcus* consortium (Su et al., 2022) detailed the Cr(VI) tolerance mechanism, showing it was mediated by the efflux of chromate via the chrA and copZ genes, supplemented by DNA-repaired proteases. For arsenic detoxification, increased expression of the arsenite oxidase enzyme was observed in *Pseudomonas* spp. AK9 and was associated with higher transformation of As(III) (Satyapal et al., 2024).

**TABLE 2:** Most frequently reported genes and enzymes involved in heavy-metal detoxification, specifying their associated metals, representative taxa, and primary function.

Gene/enzyme	Associated metals	Representative taxa	Function
chrA	Cr(VI)	*Pseudomonas* sp./*Rhodococcus* sp.	Efflux of Cr(VI)
copZ	Cr(VI)	*Pseudomonas* sp./*Rhodococcus* sp.	Cr(VI) tolerance/Efflux
Chromate reductase (ChrR) gene	Cr(VI)	*P. putida*	Cr(VI) reduction
Arsenite oxidase (enzyme)	As (arsenic)	*Pseudomonas* sp.	As(III) transformation/oxidation

Redox and enzymatic transformations were identified, respectively in 34.1% and 2.8% of the papers, particularly in *Pseudomonas* and *Bacillus* isolates (Ali et al., 2022; Sundarraj et al., 2023). Cr(VI) reduction to Cr(III) was the most frequently observed process, mediated by reductase enzymes that generate insoluble Cr(OH)3 or mixed chromite phases (Monga et al., 2022; Ye et al., 2024). Additional redox reactions included transformations between arsenic species (Ghosh et al., 2024; Satyapal et al., 2024). Enzymatic activities such as dehydrogenase, peroxidase, and superoxide dismutase were reported in 25% of studies, suggesting a coupling effect between biochemical and physicochemical detoxification (Yan et al., 2024; Wang et al., 2025).

## 4 Discussion

### 4.1 Comparative analysis of microbial performance and mechanisms

The systematic assessment of 41 studies published between 2022 and 2025 revealed a pronounced taxonomic convergence in heavy-metal bioremediation, with members of *Bacillus*, *Pseudomonas*, *Enterobacter*, and *Lysinibacillus* dominating across environmental matrices. These genera consistently exhibited high removal efficiencies for cadmium, lead, and chromium, which aligns with the robust structural and physiological traits characteristic of Gram-positive and metabolically versatile Gram-negative bacteria.

*Bacillus* species showed exceptional biosorption capacities, often exceeding 100 mg g^–1^ for Pb^2+^ and Cd^2+^ (Ye et al., 2024; Wang et al., 2025), attributable to their thick peptidoglycan layers, teichoic acids, and ureolytic biomineralization potential. Conversely, *Pseudomonas* strains, particularly *P. aeruginosa*, *P. putida*, and *P. fluorescens*, displayed remarkable multi-metal tolerance, frequently sustaining growth under 500–700 mg L^–1^ of Cd or Cr while maintaining removal rates above 70%. These outcomes are consistent with a broad detoxification spectrum in *Pseudomonas*, particularly in terms of mechanistic diversity and multi-metal tolerance, rather than uniformly higher removal efficiencies across all contexts. The predominance of these taxa corroborates prior evidence indicating that bacterial consortia combining *Bacillus*, as high-affinity sorbents, and *Pseudomonas*, as redox transformers and EPS producers, outperform single strains in multi-metal matrices (Peng et al., 2023; Cai et al., 2025).

Mixed consortia exhibited synergistic mechanisms in which *Bacillus* contributed carbonate precipitation and surface complexation, whereas *Pseudomonas* enhanced redox transformations through periplasmic reductases and metal-chelating siderophores. The co-occurrence of these functions yields a sequential detoxification process, whereby soluble ions are first immobilized extracellularly and subsequently converted to less mobile species within biofilms. This synergy underlines the ecological advantage of cooperative systems in natural and engineered environments. Three dominant pathways emerged: (i) passive biosorption through carboxyl, hydroxyl, and phosphoryl moieties within the cell wall; (ii) bioaccumulation and redox transformation mediated by enzymatic systems such as chromate reductases and arsenate reductases; and (iii) biomineralization processes involving carbonate and phosphate precipitation.

Studies employing advanced spectroscopy (FTIR, XRD, SEM–EDS) confirmed the formation of CdCO_3_, Pb_3_(PO_4_)_2_, and Cr(OH)_3_ as common end-products, providing molecular validation for immobilization mechanisms (Kaur et al., 2024; Zhang et al., 2024). Together, these findings support the interpretation that microbial metal removal is not a single process but a sequence of coupled reactions integrating physicochemical adsorption with biologically driven transformations.

### 4.2 Environmental and operational determinants of metal removal

Bioremediation performance was highly dependent on environmental conditions, particularly pH, temperature, and substrate composition. Optimal pH values clustered between 6.5 and 8.0, where deprotonation of cell-wall ligands enhances cation binding and carbonate precipitation. Acidic conditions (pH < 5) generally reduced sorption efficiency by promoting metal solubility and proton competition, although acid-tolerant *Pseudomonas* and *Rhodococcus* bacteria maintained activity in soils contaminated with mixed metals. Temperature affected both adsorption kinetics and metabolic rates, with optimal ranges between 28 °C and 35 °C across most isolates. Extreme thermophiles or psychrotolerant strains were rare, confirming that mesophilic systems dominate soil remediation under temperate climates.

Matrix effects were also significant; in fact, synthetic solutions frequently yielded higher removal percentages than soil or sludge matrices due to reduced ionic competition. However, field-relevant matrices better captured the complexity of environmental constraints and revealed a systematic reduction in remediation performance that is not evident in laboratory batch systems. In particular, studies employing real contaminated soils demonstrated that bacterial immobilization efficiency decreases by 10%–25% relative to aqueous systems, highlighting a substantial laboratory-to-field performance gap primarily due to metal–clay interactions and organic matter interference (Lin et al., 2022; Zhang et al., 2024). Nonetheless, the persistence of biofilm-forming strains may partially mitigate these losses by maintaining localized microenvironments conducive to sorption and precipitation, although it does not fully offset the observed laboratory-to-field performance gap.

Nutrient availability and carbon sources further modulated bioremediation kinetics. Simple sugars and organic acids facilitated EPS synthesis, enhancing sorption sites and metal chelation. The inclusion of urea or ammonium salts in media triggered MICP, increasing immobilization through CaCO_3_ lattice formation. Such findings suggest that bioremediation efficiency arises from a balance between microbial physiology and physicochemical context, where even minor shifts in pH or nutrient status can redefine metal speciation and bioavailability.

While *Bacillus* species remain among the most efficient biosorbents due to their robust cell walls and sporulation capacity, recent evidence highlights the broader ecological and mechanistic versatility of *Pseudomonas* spp. as model organisms and reference systems for heavy-metal bioremediation. However, the application of *Pseudomonas* in field-scale remediation is constrained by several operational limitations. As non-spore-forming bacteria, *Pseudomonas* strains generally display reduced long-term persistence under desiccation, UV exposure, nutrient limitation, and fluctuating redox conditions when compared with spore-forming genera such as *Bacillus*. These constraints often necessitate repeated inoculation, immobilization on carriers (e.g., biochar, alginate, mineral matrices), or integration into biofilm-supported systems to ensure sustained activity in soil environments. Comparative analyses indicate that *Pseudomonas* can operate effectively across a wide spectrum of environmental conditions, including acidic and multi-metal-contaminated soils, where *Bacillus* may exhibit reduced metabolic activity, depending on strain-specific and matrix-related factors. Genomically, *Pseudomonas* is shown in the dataset to harbor a well-characterized set of resistance operons, such as chromate efflux systems (e.g., chrA) and specific reductases (Su et al., 2022; Sundarraj et al., 2023), along with stress-response regulators under quorum-sensing control (Lu et al., 2022), which may confer rapid adaptability and multi-metal tolerance. From an applicative standpoint, non-spore-forming *Pseudomonas* strains develop resilient biofilms capable of maintaining metabolic activity under fluctuating redox and moisture conditions, making them suitable for continuous bioreactors and biosensor interfaces. Moreover, the genus is one of the most genomically annotated among soil bacteria, providing a reproducible framework for cross-study comparison and systems-biology modeling. Overall, these observations indicate that removal efficiencies reported under controlled laboratory conditions tend to overestimate field performance, underscoring the need for cautious extrapolation and for validation under realistic soil and environmental conditions.

### 4.3 Functional and genetic determinants in *Pseudomonas* spp.

Within the analyzed dataset, *Pseudomonas* spp. consistently represented the most functionally diverse genus. Crucially, it also stands out as one of the most mechanistically and genetically well-characterized genera in the context of these studies, providing a clearer and more reproducible framework for its application compared to many other taxa.

Genome-level investigations and specific gene analyses, which were reported for *Pseudomonas* more frequently than for other genera, have begun to elucidate its extensive repertoire of resistance determinants. For example, specific genomic analyses in the dataset linked Cr(VI) tolerance directly to the function of efflux genes chrA and copZ (Su et al., 2022). This was further supported by studies isolating the specific chromate reductase (ChrR) gene from *P. putida*, supporting its role in Cr(VI) reduction (Sundarraj et al., 2023). Similarly, arsenic detoxification was explicitly correlated with the “increased expression of arsenite oxidase” in *Pseudomonas* sp. (Satyapal et al., 2024).

This depth of available genetic information—spanning efflux systems, enzymatic reduction, and stress-response enzymes—is complemented by its proven metabolic versatility. Mechanistically, *Pseudomonas* exhibits a dual detoxification strategy that combines:

1. Passive biosorption: via copious EPS and biofilm matrices rich in functional groups.
2. Active metabolic reduction: (e.g., Cr(VI) to Cr(III)) mediated by the reductase enzymes localized in the cytoplasmic membrane.

While other genera, particularly *Bacillus*, often demonstrate exceptionally high removal efficiencies through passive biosorption, biomineralization, and long-term environmental persistence, *Pseudomonas* spp. are better interpreted as a mechanistic benchmark rather than a universally superior remediation agent. The value of *Pseudomonas* lies primarily in the depth of available genetic, transcriptomic, and biochemical characterization, which enables detailed dissection of efflux systems, redox enzymes, and regulatory networks that are less frequently resolved in other taxa.

The combination of its broad metabolic capabilities (including redox transformations often lacking in other biosorbents), its detailed genomic annotation, and its robust stress-response mechanisms makes it a particularly well suited and adaptable candidate for bioaugmentation, depending on the environmental context and remediation objectives.

### 4.4 Integrative interpretation and ecological implications

The evidence underscores that microbial bioremediation is a multi-layered process integrating community ecology, metal chemistry, and environmental dynamics. In terrestrial matrices, microbial assemblages restructure in response to chronic metal exposure, selecting for resistant phenotypes with enhanced EPS and biofilm production. This adaptive remodeling not only supports metal immobilization but also contributes to soil stabilization and nutrient cycling. The co-occurrence of plant growth promoting traits, such as phosphate solubilization, indole-acetic-acid synthesis, and siderophore production, demonstrates that many remediation-competent strains simultaneously enhance soil fertility, bridging the gap between bioremediation and agroecological restoration (Gao et al., 2024; Ghosh et al., 2024). From an applied perspective, the convergence of adsorption, biomineralization, and redox conversion mechanisms across different taxa suggests that integrated consortia or sequential treatment systems can achieve superior outcomes in heterogeneous contaminated soils. Field-scale trials incorporating *Pseudomonas–Bacillus* co-inocula or biochar-supported bacterial systems have demonstrated stable performance under fluctuating pH and moisture. However, ensuring ecological compatibility and long-term persistence remains essential to avoid unintended perturbations of indigenous microbiota. At the same time, the environmental sustainability of microbial remediation extends beyond removal efficiency. The re-establishment of soil microbial diversity and enzymatic functionality is crucial to restoring ecological services after decontamination. The capacity of *Pseudomonas* to reinstate biogeochemical cycling reinforces the dual role of these bacteria as both detoxifiers and ecological engineers.

### 4.5 Limitations and perspectives for future research

Although the present synthesis provides robust evidence on the efficiency and mechanisms of bacterial heavy-metal remediation, several methodological limitations persist. The heterogeneity of experimental conditions, particularly regarding initial metal concentrations, exposure times, and analytical protocols, still hampers quantitative comparability among studies. Many works rely on batch experiments with synthetic solutions, which tend to overestimate removal rates compared with complex soil matrices. Moreover, the limited number of field-scale investigations constrains the extrapolation of laboratory findings to real environments.

Despite the rigorous application of PRISMA inclusion criteria and NOS quality assessment, several potential sources of bias remain inherent to the analyzed literature. These include selection bias arising from the restriction to English-language publications and recent years (2022–2025), experimental and analytical heterogeneity related to differences in matrix type, initial metal concentrations, exposure duration, and analytical techniques (AAS, ICP-OES, ICP-MS), as well as matrix bias associated with the frequent use of synthetic batch systems that tend to overestimate removal efficiencies relative to field conditions. Additional reporting bias may result from the inconsistent disclosure of replicate numbers, standard deviations, or negative outcomes, while interpretation bias can occur when the presence of resistance genes (e.g., *chrA*, *copZ*, *arsC*) is equated with functional detoxification in the absence of enzymatic or transcriptional validation.

A synopsis of the potential sources of bias is in the Supplementary Table 7, and according to authors’ point of view there are at least five different sources/kinds of bias (matrix, performance, analytical, reporting, and mechanistic), each of them characterized by low, medium, and high levels of risk.

Addressing these limitations will require a shift toward integrated, functionally validated research frameworks. Future studies should prioritize whole-genome and transcriptomic approaches to resolve regulatory networks underlying metal resistance and to support predictive modeling of microbial community dynamics. In parallel, hybrid biotechnological systems combining microbial consortia with immobilization strategies (e.g., biochar, alginate carriers, or plant-assisted platforms) should be further explored to enhance stability, persistence, and ecological compatibility under field conditions. Emerging tools, including AI-assisted strain selection and CRISPR-based gene editing, offer promising avenues to validate and fine-tune key efflux and redox pathways, improving both specificity and kinetics of metal transformation. Long-term, field-based monitoring integrated with digital environmental sensing will be essential to assess persistence, horizontal gene transfer, and ecosystem resilience, thereby enabling the transition from laboratory-scale performance to scalable, data-driven, and environmentally sustainable bioremediation systems.

## 5 Conclusion

Microbial bioremediation of heavy metals emerges as a multifactorial process governed by genetic, biochemical, and ecological interactions. Across the 41 studies analyzed, a consistent pattern highlights the dominance of *Pseudomonas* and *Bacillus* species as functional pillars of detoxification, supported by complementary mechanisms such as EPS-mediated biosorption, enzymatic redox transformations, and biomineralization. Among these, *Pseudomonas* stands out as a benchmark genus for its genomic plasticity, multi-metal tolerance, and biofilm-mediated resilience under variable environmental conditions.

The integration of these microbial traits with advanced analytical, genomic, and digital tools will enable the transition from laboratory-scale experiments to sustainable field applications. Future developments should therefore focus on standardized methodologies, long-term ecological monitoring, and the incorporation of predictive models to ensure the stability and scalability of microbially driven soil restoration.

Advanced microbial bioremediation will require the convergence of microbiology, systems biology, and environmental engineering toward a unified, data-driven framework for soil and water detoxification. Harnessing the inherent adaptability of microbial consortia, particularly *Pseudomonas*-based communities, while embedding sustainability, biosafety, and ethical governance principles will be crucial to translate current research into resilient, climate-responsive solutions. Through this integrative approach, microbial biotechnology can evolve from a remediation tool into a cornerstone of ecological restoration and circular-economy strategies.

## Data Availability

All data analyzed including strings, and the articles selected for this review, are provided in the online Supplementary material accompanying this manuscript.
