# Supplementary material for: PRISMA-based review of Pseudomonas spp. in microbial heavy-metal bioremediation: mechanisms and taxonomy
Source: Front Microbiol. 2026 Feb 27;17:1760258. doi: 10.3389/fmicb.2026.1760258 (PMC12982460; doi:10.3389/fmicb.2026.1760258)
Supplement: Supplementary file 1 [file Table_1.docx]

Supplementary Material

**Supplementary Table S1** PRISMA 2020 checklist summarizing compliance of the present systematic review with reporting standards

| **Section / Topic** | **Item #** | **Checklist Item (abridged)** | **Location in manuscript** |
| --- | --- | --- | --- |
| **TITLE** | 1 | Identify the report as a systematic review. | Title page + Abstract line 1 |
| **ABSTRACT** | 2 | Provide a structured summary including background, objectives, data sources, eligibility criteria, results, and conclusions. | Abstract |
| **INTRODUCTION** | 3 | Describe the rationale for the review. | Section 1 (Introduction, lines 1-10) |
|  | 4 | State the objectives or research questions. | Section 1, final paragraph |
| **METHODS** | 5 | Specify inclusion and exclusion criteria. | Section 2.3 + Supp. Table S2 |
|  | 6 | Specify all information sources (databases, registers). | Section 2.2 |
|  | 7 | Present full search strategy for at least one database. | Section 2.2 + Supp. File (Query .docx) |
|  | 8 | Describe the selection process. | Section 2.4 |
|  | 9 | Describe data-collection process. | Section 2.5 |
|  | 10 | Describe data items (variables extracted). | Section 2.5 |
|  | 11 | Describe methods used to assess risk of bias or study quality. | Section 2.6 |
|  | 12 | Specify effect-measures or summary measures used. | Section 2.8 |
|  | 13 | Describe synthesis methods and any subgroup or sensitivity analyses. | Section 2.8 |
|  | 14 | Describe how certainty or confidence in the evidence was assessed. | Section 2.6 |
| **RESULTS** | 16a | Describe results of search and selection (ideally with a flow diagram). | Section 2.4 + Figure 1 |
|  | 16b | Cite reasons for exclusion at each stage. | Section 2.4 + Supp. Table S2 |
|  | 17 | Present characteristics of included studies. | Section 3.1 + Section 3.2 |
|  | 18 | Present results of individual studies. | Results section 3.1–3.4 |
|  | 19 | Present synthesis of results. | Results & Discussion |
| **DISCUSSION** | 23 | Summarize main findings, limitations, implications. | Section 4 |
|  | 24 | Discuss limitations of evidence and of the review process. | Section 4.5 |
|  | 25 | Provide a general interpretation in the context of other evidence. | Section 5 |
| **OTHER INFORMATION** | 26 | Describe registration or protocol information. | Not applicable |
|  | 27 | Report sources of funding and role of funders. | Declarations |
|  | 28 | Report competing interests. | Declarations |
|  | 29 | Report data availability and supplementary materials. | Declarations |

**Supplementary Table S2** Resume of research strings used per each platform and obtained results

| **Scientific platform** | **Research query** | **Results** |
| --- | --- | --- |
| **Scopus** | 1. TITLE-ABS-KEY ("bioremediation" AND ("heavy metals" OR cadmium OR lead OR chromium OR nickel OR mercury OR arsenic OR copper OR zinc)) AND PUBYEAR > 2000 | **34.230** |
| **Scopus** | 1. TITLE-ABS-KEY ("microbiota" OR "bacteria" OR "microorganism*") AND TITLE-ABS-KEY ("heavy metals" OR cadmium OR lead OR chromium OR nickel OR mercury OR arsenic OR copper OR zinc) AND PUBYEAR > 2000 | **149.001** |
| **Scopus** | 1. TITLE-ABS-KEY ( microbial OR bacteria OR microorganism* OR microbiota ) AND TITLE-ABS-KEY ( "bioremediation" ) AND TITLE-ABS-KEY ( "heavy metals" OR cadmium OR lead OR chromium OR nickel OR mercury OR arsenic OR copper OR zinc ) AND PUBYEAR > 2000 AND ( LIMIT-TO ( SUBJAREA , "ENVI" ) OR LIMIT-TO ( SUBJAREA , "BIOC" ) OR LIMIT-TO ( SUBJAREA , "IMMU" ) OR LIMIT-TO ( SUBJAREA , "AGRI" ) ) | **11.547** |
| **Scopus** | 1. TITLE ( microbial OR bacteria OR microorganism* OR microbiota ) AND TITLE ( "bioremediation" ) AND TITLE ( "heavy metals" OR cadmium OR lead OR chromium OR nickel OR mercury OR arsenic OR copper OR zinc ) AND NOT TITLE-ABS-KEY ( aquaculture OR marine OR dye* OR textile* OR "organic pollutant*" OR "nanoparticle*" OR "rare earth*" OR "photocatalys*" OR "antibiotic*" ) AND PUBYEAR > 2020 AND ( LIMIT-TO ( SUBJAREA,"ENVI" ) OR LIMIT-TO ( SUBJAREA,"BIOC" ) OR LIMIT-TO ( SUBJAREA,"IMMU" ) OR LIMIT-TO ( SUBJAREA,"AGRI" ) ) | **315** |
| **Scopus** | 1. TITLE-ABS-KEY ( Pseudomonas OR Bacillus OR Rhodococcus OR Acinetobacter OR Shewanella OR Geobacter ) AND TITLE-ABS-KEY ( microbial OR bacteria OR microorganism* OR microbiota ) AND TITLE-ABS-KEY ( "bioremediation" ) AND TITLE-ABS-KEY ( "heavy metals" OR cadmium OR lead OR chromium OR nickel OR mercury OR arsenic OR copper OR zinc ) AND NOT TITLE-ABS-KEY ( phytoremediation OR aquaculture OR dye* OR textile* OR "organic pollutant*" OR "nanoparticle*" OR "rare earth*" OR "photocatalys*" OR "antibiotic*" ) AND PUBYEAR > 2022 AND ( LIMIT-TO ( SUBJAREA , "ENVI" ) OR LIMIT-TO ( SUBJAREA , "BIOC" ) OR LIMIT-TO ( SUBJAREA , "IMMU" ) OR LIMIT-TO ( SUBJAREA , "AGRI" ) ) AND ( LIMIT-TO ( DOCTYPE , "ar" ) OR LIMIT-TO ( DOCTYPE , "re" ) ) AND ( LIMIT-TO ( PUBYEAR , 2022 ) OR LIMIT-TO ( PUBYEAR , 2023 ) OR LIMIT-TO ( PUBYEAR , 2024 ) OR LIMIT-TO ( PUBYEAR , 2025 ) ) | **825** |
| **Scopus** | 1. TITLE-ABS-KEY ( Pseudomonas OR Bacillus OR Rhodococcus OR Acinetobacter OR Shewanella OR Geobacter ) AND TITLE-ABS-KEY ( microbial OR bacteria OR microorganism* OR microbiota ) AND TITLE-ABS-KEY ( soil OR dirt ) AND TITLE-ABS-KEY ( "bioremediation") AND TITLE-ABS-KEY ( "heavy metal*" OR cadmium OR lead OR chromium OR nickel OR mercury OR arsenic OR copper OR zinc ) AND TITLE-ABS-KEY ( biosorption OR bioaccumulation OR biomineralization OR bioprecipitation OR "redox transformation" ) AND NOT TITLE-ABS-KEY ( phytoremediation OR phytoremediation OR aquaculture OR dye* OR textile* OR "organic pollutant*" OR "nanoparticle*" OR "rare earth*" OR "photocatalys*" OR "antibiotic*" ) AND PUBYEAR > 2022 AND ( LIMIT-TO ( SUBJAREA,"ENVI" ) OR LIMIT-TO ( SUBJAREA,"BIOC" ) OR LIMIT-TO ( SUBJAREA,"IMMU" ) OR LIMIT-TO ( SUBJAREA,"AGRI" ) ) AND ( LIMIT-TO ( DOCTYPE,"ar" ) OR LIMIT-TO ( DOCTYPE,"re" ) ) AND ( LIMIT-TO ( PUBYEAR,2022) OR LIMIT-TO ( PUBYEAR,2023) OR LIMIT-TO ( PUBYEAR,2024) OR LIMIT-TO ( PUBYEAR,2025) ) | **98** |
| **Web of Science** | 1. TS=( "bioremediation" AND ("heavy metals" OR cadmium OR lead OR chromium OR nickel OR mercury OR arsenic OR copper OR zinc) ) AND PY=(2001-2025) | **11.646** |
| **Web of Science** | 1. TS=( ("microbiota" OR bacteria OR microorganism*) AND ("heavy metals" OR cadmium OR lead OR chromium OR nickel OR mercury OR arsenic OR copper OR zinc) ) AND PY=(2001-2025) | **161.040** |
| **Web of Science** | 1. TS=( (microbial OR bacteria OR microorganism* OR microbiota) AND bioremediation AND ("heavy metals" OR cadmium OR lead OR chromium OR nickel OR mercury OR arsenic OR copper OR zinc) ) AND PY=(2001-2025) | **7.529** |
| **Web of Science** | 1. TI=( microbial OR bacteria OR microorganism* OR microbiota ) AND TI=( bioremediation ) AND TI=( "heavy metals" OR cadmium OR lead OR chromium OR nickel OR mercury OR arsenic OR copper OR zinc ) NOT TS=( aquaculture OR marine OR dye* OR textile* OR "organic pollutant*" OR nanoparticle* OR "rare earth*" OR photocatalys* OR antibiotic* ) AND PY=(2021-2025) | **86** |
| **Web of Science** | 1. TS=( (Pseudomonas OR Bacillus OR Rhodococcus OR Acinetobacter OR Shewanella OR Geobacter) AND (microbial OR bacteria OR microorganism* OR microbiota) AND bioremediation AND ("heavy metals" OR cadmium OR lead OR chromium OR nickel OR mercury OR arsenic OR copper OR zinc) ) NOT TS=( phytoremediation OR aquaculture OR dye* OR textile* OR "organic pollutant*" OR nanoparticle* OR "rare earth*" OR photocatalys* OR antibiotic* ) AND PY=(2022-2025) | **636** |
| **Web of Science** | 1. TS=( (Pseudomonas OR Bacillus OR Rhodococcus OR Acinetobacter OR Shewanella OR Geobacter) AND (microbial OR bacteria OR microorganism* OR microbiota) AND (soil OR dirt) AND bioremediation AND ("heavy metal*" OR cadmium OR lead OR chromium OR nickel OR mercury OR arsenic OR copper OR zinc) AND (biosorption OR bioaccumulation OR biomineralization OR bioprecipitation OR "redox transformation") ) NOT TS=( phytoremediation OR aquaculture OR dye* OR textile* OR "organic pollutant*" OR nanoparticle* OR "rare earth*" OR photocatalys* OR antibiotic* ) AND PY=(2022-2025) | **86** |
| **PubMed** | 1. (bioremediation[MeSH Terms] OR bioremediation[Title/Abstract]) AND ("heavy metals"[MeSH Terms] OR "heavy metals"[Title/Abstract] OR cadmium[Title/Abstract] OR lead[Title/Abstract] OR chromium[Title/Abstract] OR nickel[Title/Abstract] OR mercury[Title/Abstract] OR arsenic[Title/Abstract] OR copper[Title/Abstract] OR zinc[Title/Abstract])AND ("2000/01/01"[Date - Publication] : "2025/12/31"[Date - Publication]) | **11.271** |
| **PubMed** | 1. ("microbiota"[MeSH Terms] OR microbiota[Title/Abstract] OR bacteria[Title/Abstract] OR microorganism*[Title/Abstract]) AND ("heavy metals"[MeSH Terms] OR "heavy metals"[Title/Abstract] OR cadmium[Title/Abstract] OR lead[Title/Abstract] OR chromium[Title/Abstract] OR nickel[Title/Abstract] OR mercury[Title/Abstract] OR arsenic[Title/Abstract] OR copper[Title/Abstract] OR zinc[Title/Abstract]) AND ("2000/01/01"[Date - Publication] : "2025/12/31"[Date - Publication]) | **43.915** |
| **PubMed** | 1. (microbial[Title/Abstract] OR bacteria[Title/Abstract] OR microorganism*[Title/Abstract] OR microbiota[Title/Abstract]) AND (bioremediation[MeSH Terms] OR bioremediation[Title/Abstract]) AND ("heavy metals"[MeSH Terms] OR "heavy metals"[Title/Abstract] OR cadmium[Title/Abstract] OR lead[Title/Abstract] OR chromium[Title/Abstract] OR nickel[Title/Abstract] OR mercury[Title/Abstract] OR arsenic[Title/Abstract] OR copper[Title/Abstract] OR zinc[Title/Abstract])AND ("2000/01/01"[Date - Publication] : "2025/12/31"[Date - Publication]) | **4.230** |
| **PubMed** | 1. (microbial[Title] OR bacteria[Title] OR microorganism*[Title] OR microbiota[Title]) AND (bioremediation[Title]) AND ("heavy metals"[Title] OR cadmium[Title] OR lead[Title] OR chromium[Title] OR nickel[Title] OR mercury[Title] OR arsenic[Title] OR copper[Title] OR zinc[Title]) NOT (aquaculture[Title/Abstract] OR marine[Title/Abstract] OR dye*[Title/Abstract] OR textile*[Title/Abstract] OR "organic pollutant*"[Title/Abstract] OR nanoparticle*[Title/Abstract] OR "rare earth*"[Title/Abstract] OR photocatalys*[Title/Abstract] OR antibiotic*[Title/Abstract]) AND ("2021/01/01"[Date - Publication] : "2025/12/31"[Date - Publication]) | **47** |
| **PubMed** | 1. (Pseudomonas[MeSH Terms] OR Bacillus[MeSH Terms] OR Rhodococcus[Title/Abstract] OR Acinetobacter[MeSH Terms] OR Shewanella[MeSH Terms] OR Geobacter[MeSH Terms] OR Pseudomonas[Title/Abstract] OR Bacillus[Title/Abstract] OR Acinetobacter[Title/Abstract] OR Shewanella[Title/Abstract] OR Geobacter[Title/Abstract]) AND (microbial[Title/Abstract] OR bacteria[Title/Abstract] OR microorganism*[Title/Abstract] OR microbiota[Title/Abstract]) AND (bioremediation[MeSH Terms] OR bioremediation[Title/Abstract]) AND ("heavy metals"[MeSH Terms] OR "heavy metals"[Title/Abstract] OR cadmium[Title/Abstract] OR lead[Title/Abstract] OR chromium[Title/Abstract] OR nickel[Title/Abstract] OR mercury[Title/Abstract] OR arsenic[Title/Abstract] OR copper[Title/Abstract] OR zinc[Title/Abstract]) NOT (phytoremediation[Title/Abstract] OR aquaculture[Title/Abstract] OR dye*[Title/Abstract] OR textile*[Title/Abstract] OR "organic pollutant*"[Title/Abstract] OR nanoparticle*[Title/Abstract] OR "rare earth*"[Title/Abstract] OR photocatalys*[Title/Abstract] OR antibiotic*[Title/Abstract]) AND ("2022/01/01"[Date - Publication] : "2025/12/31"[Date - Publication]) | **74** |
| **PubMed** | 1. (Pseudomonas[Title/Abstract] OR Bacillus[Title/Abstract] OR Rhodococcus[Title/Abstract] OR Acinetobacter[Title/Abstract] OR Shewanella[Title/Abstract] OR Geobacter[Title/Abstract])AND (microbial[Title/Abstract] OR bacteria[Title/Abstract] OR microorganism*[Title/Abstract] OR microbiota[Title/Abstract]) AND (soil[Title/Abstract] OR dirt[Title/Abstract]) AND (bioremediation[MeSH Terms] OR bioremediation[Title/Abstract]) AND ("heavy metals"[MeSH Terms] OR "heavy metals"[Title/Abstract] OR cadmium[Title/Abstract] OR lead[Title/Abstract] OR chromium[Title/Abstract] OR nickel[Title/Abstract] OR mercury[Title/Abstract] OR arsenic[Title/Abstract] OR copper[Title/Abstract] OR zinc[Title/Abstract]) AND (biosorption[Title/Abstract] OR bioaccumulation[Title/Abstract] OR biomineralization[Title/Abstract] OR bioprecipitation[Title/Abstract] OR "redox transformation"[Title/Abstract]) NOT (phytoremediation[Title/Abstract] OR aquaculture[Title/Abstract] OR dye*[Title/Abstract] OR textile*[Title/Abstract] OR "organic pollutant*"[Title/Abstract] OR nanoparticle*[Title/Abstract] OR "rare earth*"[Title/Abstract] OR photocatalys*[Title/Abstract] OR antibiotic*[Title/Abstract]) AND ("2022/01/01"[Date - Publication] : "2025/12/31"[Date - Publication]) | **4** |

**Supplementary Table S3** Eligibility criteria and rationale for inclusion and exclusion applied in the systematic review. The table details the methodological boundaries used to determine study eligibility, including study type, biological system, target metals, environmental matrices, quantitative evidence, analytical validation, taxonomic identification, and mechanistic requirements. Criteria were established a priori in accordance with the PRISMA 2020 guidelines to ensure transparency, reproducibility, and methodological consistency across the study selection process.

| **Category** | **Inclusion criteria** | **Exclusion criteria / rationale for exclusion** |
| --- | --- | --- |
| **Study type and publication features** | Original experimental research articles published in peer-reviewed journals (2022–2025). Written in English. Contain reproducible methodology and quantitative results. | Review, mini-review, opinion, or conceptual papers without new data. Book chapters, proceedings, or non-peer-reviewed publications. Duplicate or inaccessible full texts. |
| **Biological system** | Microbial bioremediation in which bacteria, actinomycetes, or mixed microbial consortia represent the primary remediation agents. | Plant-centered phytoremediation or plant–microbe synergy where the main mechanism is plant-based. Purely abiotic or enzymatic systems lacking living cells. |
| **Target pollutants** | Experiments addressing at least one toxic heavy metal (Cd, Pb, Cr, Ni, Cu, Zn, Hg, As). | Studies limited to non-metal pollutants (dyes, hydrocarbons, antibiotics, microplastics, pesticides, organics, rare-earths). |
| **Environmental matrix** | Environmentally relevant matrices: soil, sediment, wastewater, effluent, sludge, or simulated soil systems. | Aquatic or laboratory systems with no soil relevance (e.g., pure-culture in liquid medium without environmental context). |
| **Quantitative evidence required** | At least one measurable outcome: metal-removal percentage, adsorption capacity (qₘₐₓ), ecc. | Only qualitative or descriptive observations with no numerical metrics. |
| **Analytical validation** | Robust analytical techniques: AAS, ICP-OES/ICP-MS, XRF, SEM/EDS, FTIR, XRD, or equivalent. | Indirect colorimetric or unvalidated assays lacking instrumental confirmation. |
| **Taxonomic identification** | Identification to at least the genus level (preferably 16S rRNA-based or WGS). | Unidentified microbial sources or unclassified mixed cultures. |
| **Mechanistic or molecular characterization** | Presence of biochemical or genetic evidence supporting detoxification (EPS, biofilm, enzymes, siderophores, biomineralization, resistance genes. | Absence of mechanistic data or purely descriptive tolerance assays. |
| **Metagenomic and omics studies** | Included only when quantitative gene/pathway data directly linked to metal detoxification or resistance are reported. | Omics studies lacking quantitative association between genes and detoxification performance. |

**Supplementary Table S4** NOS assessment of the included works for review according to PRISMA 2020 protocol.

| **Author (year)** | **Title** | **Journal** | **NOS score** |
| --- | --- | --- | --- |
| Wang et al., 2025 | Lead biosorption by *Bacillus tequilensis* FB-6: characterization of resistance mechanisms and remediation potential | Journal of the Science of Food and Agriculture | 9 |
| Kaushal and Pati, 2024 | *Bacillus altitudinis* Mediated Lead Bioremediation for Enhanced Growth of Rice Seedlings | Current Microbiology | 8 |
| Kashyap et al., 2022 | Biosorption efficiency of nickel by various endophytic bacterial strains for removal of nickel from electroplating industry effluents: an operational study | Ecotoxicology | 8 |
| Mtengai et al., 2022 | Existence of a novel heavy metal–tolerant *Pseudomonas aeruginosa* strain Zambia SZK-17 Kabwe 1: the potential bioremediation agent in the heavy metal–contaminated area | Environmental Monitoring and Assessment | 7 |
| Henagamage et al., 2022 | Fungal-bacterial biofilm mediated heavy metal rhizo-remediation | World Journal of Microbiology and Biotechnology | 8 |
| Ye et al., 2024 | Mechanism of Cr(VI) removal by efficient Cr(VI)-resistant *Bacillus mobilis* CR3 | World Journal of Microbiology and Biotechnology | 9 |
| Monga et al., 2022 | solation and Identification of Novel Chromium Tolerant Bacterial Strains From a Heavy Metal Polluted Urban Creek: An Assessment of Bioremediation Efficiency and Flocculant Production | Thalassas: International Journal of Marine Sciences | 8 |
| Shaaban Emara et al., 2023 | Mechanistic action of lead removal by the native isolate *Bacillus amyloliquefaciens* ON261680.1 | Arabian Journal of Chemistry | 9 |
| Ghosh et al., 2024 | Plant growth-promoting *Bacillus cereus* MCC3402 facilitates rice seedling growth under arsenic-spiked soil | Biocatalysis and Agricultural Biotechnology | 8 |
| Sahu et al., 2022 | Bacterial strains found in the soils of a municipal solid waste dumping site facilitated phosphate solubilization along with cadmium remediation | Chemosphere | 8 |
| Ali et al., 2022 | Cadmium tolerant microbial strains possess different mechanisms for cadmium biosorption and immobilization in rice seedlings | Chemosphere | 8 |
| Lin et al., 2022 | Construction of bifunctional bacterial community for co-contamination remediation: Pyrene biodegradation and cadmium biomineralization | Chemosphere | 9 |
| Zuo et al., 2022 | Using *Bacillus thuringiensis* HM-311 hydroxyapatite biochar beads to remediate Pb and Cd contaminated farmland soil | Chemosphere | 9 |
| Gao et al., 2024 | Contribution of Cd passivating functional bacterium H27 to tobacco growth under Cd stress | Chemosphere | 8 |
| Kaur et al., 2024 | Removal of cadmium through biomineralization using halophilic and ureolytic bacteria under saline conditions | International Biodeterioration & Biodegradation | 9 |
| Soto-Ramírez et al., 2024 | Engineering the cell wall reactive groups of Plant Growth Promoting Rhizobacteria by culture strategy for heavy metal removal. | Journal of Biotechnology | 9 |
| Mondal et al., 2025 | Bioprotective mechanisms of *Enterobacter* sp. against arsenic, cadmium, and lead toxicity and its potential role in soil bioremediation. | Journal of Environmental Chemical Engineering | 9 |
| Huang et al., 2024 | Immobilization of Cd2+ in an aqueous environment using a two-step microbial-induced carbonate precipitation method. | Journal of Environmental Management | 8 |
| Yan et al., 2024 | Cadmium biosorption and mechanism investigation using two cadmium-tolerant microorganisms isolated from rhizosphere soil of rice | Journal of Hazardous Materials | 9 |
| Cai et al., 2025 | A cooperation mechanism between *Bacillus thuringiensis* and *Citrobacter freundii* that enhances cadmium biomineralization | Journal of Hazardous Materials | 9 |
| Chen et al., 2025 | Integrated analyses of characterization and transcriptome reveal the adaptive response mechanism of *Bacillus cereus* FCHN 7–1 in cadmium adsorption | Journal of Hazardous Materials | 8 |
| Peng et al., 2023 | Mixed bacteria passivation for the remediation of arsenic, lead, and cadmium: Medium optimization and mechanisms. | Process Safety and Environmental Protection | 9 |
| Su et al., 2022 | Characterization of the simultaneous degradation of pyrene and removal of Cr(VI) by a bacteria consortium YH | Science of the Total Environment | 9 |
| Zhang et al., 2023 | Cadmium-tolerant *Bacillus cereus* 2–7 alleviates the phytotoxicity of cadmium exposure in banana plantlets | Science of the Total Environment | 9 |
| Zhang et al., 2024a | Bioremediation of paddy soil with amphitropic mixture markedly attenuates rice cadmium: Effect of soil cadmium removal and Fe/S-cycling bacteria in rhizosphere | Science of the Total Environment | 9 |
| Zhang et al., 2024b | Biogenic calcium improved Cd2+ and Pb2+ immobilization in soil using the ureolytic bacteria *Bacillus pasteurii*. | Science of the Total Environment | 9 |
| Elahi et al., 2022 | Isolation and characterization of a highly effective bacterium *Bacillus cereus b-525k* for hexavalent chromium detoxification | Saudi Journal of Biological Sciences | 8 |
| Mitra et al., 2025 | Halotolerant bacteria isolated from the soils of Indian mangrove ecosystem for metal removal and NPK enhancement. | Scientific Reports | 9 |
| Manikandan and Nair, 2023 | Developing a biocatalyst showcasing the synergistic effect of rice husk biochar and bacterial cells for the removal of heavy metals. | New Journal of Chemistry | 8 |
| Shan et al., 2024 | Bioremediation Potential of Cr(VI) by *Lysinibacillus cavernae* CR-2 Isolated from Chromite-Polluted Soil: A Promising Approach for Cr(VI) Detoxification. | Geomicrobiology Journal | 9 |
| Surabhi et al., 2025 | Multi-metal Tolerance Efficiency and Bioremediation Capabilities of *Bacillus aerius* from Mangrove Rhizosphere | Geomicrobiology Journal | 9 |
| Satyapal et al., 2024 | Gamma irradiation in modulating arsenic bioremediation potential of *Pseudomonas* sp. AK1 and AK9 | International Journal of Radiation Biology |  |
| Bhandari et al., 2024 | Microbial bioremediation: unraveling the potential for cleaner environments through comparative analysis | Bioremediation Journal | 8 |
| Sundarraj et al., 2023 | Bioremediation of hexavalent chromium by transformation of *Escherichia coli* DH5α with chromate reductase (ChrR) genes of *Pseudomonas putida* isolated from tannery effluent | Journal of Applied Microbiology | 8 |
| Yasmin et al., 2022 | Biosorptive Potential of *Pseudomonas* species RY12 Toward Zinc Heavy Metal in Agriculture Soil Irrigated with Contaminated Waste Water | Dose–Response | 8 |
| Sizentsov et al., 2025 | In vitro assessment of the biosorption potential of some representatives of тне genus bacillus during interaction with lead cations | Applied Ecology and Environmental Research | 7 |
| Liang et al., 2025 | Isolation and characterization of cadmium-resistant *Bacillus cereus* strains from Cd-contaminated mining areas for potential bioremediation applications | Frontiers in Microbiology | 9 |
| Firincă et al., 2023 | Microbial Removal of Heavy Metals from Contaminated Environments Using Metal-Resistant Indigenous Strains | Journal of Xenobiotics | 9 |
| Arce-Inga et al., 2022 | Bioremediation Potential of Native *Bacillus* sp. Strains as a Sustainable Strategy for Cadmium Accumulation of Theobroma cacao in Amazonas Region | Microorganisms | 9 |
| Mungla et al., 2022 | Assessing the Potential of Mechanical Aeration Combined with Bioremediation Process in Soils and Coastal Sediments Impacted by Heavy Metals Bioremediation Process in Soils and Coastal Sediments Impacted by  Heavy Metals | AIMS Environmental Science | 8 |
| Dahnoun et al., 2024 | Characterization and bioremediation potential of heavy-metal resistant bacteria isolated from agricultural soil | Turkish Journal of Agriculture and Forestry | 9 |

**Supplementary Table S5** NOS assessment of the included works for review according to PRISMA 2020 protocol. Stratification for microorganisms and metals.

| **Genus** | **Target metal** | **No. of studies (n)** | **Mean NOS score** | **Main matrix type** |
| --- | --- | --- | --- | --- |
| *Pseudomonas* | Cr(VI) | 8 | 8.4 | Lab + soil |
| *Pseudomonas* | Cd | 5 | 7.8 | Soil |
| *Bacillus* | Pb | 10 | 7.1 | Mainly soil |
| *Bacillus* | Cd | 7 | 7.0 | Soil |
| *Enterobacter* | Cd / Pb | 4 | 7.6 | Lab |
| Mixed consortia | Multi-metal | 7 | 8.2 | Lab + soil |

**Supplementary Table S6** Top 50 keywords identified by VOSviewer in the literature on microbial heavy-metal bioremediation (2020–2025). Keywords are ranked by occurrence and total link strength.

| **keyword** | **occurrences** | **total link strength** |
| --- | --- | --- |
| bioremediation | 10427 | 253412 |
| biodegradation, environmental | 4373 | 134323 |
| metabolism | 3626 | 111758 |
| heavy metal | 3523 | 102341 |
| bacteria | 3523 | 101981 |
| bacterium | 3514 | 106733 |
| controlled study | 3267 | 109929 |
| heavy metals | 2964 | 74765 |
| soil pollution | 2886 | 88036 |
| chemistry | 2537 | 84746 |
| soil pollutants | 2389 | 81450 |
| cadmium | 2298 | 73013 |
| soil pollutant | 2258 | 77430 |
| microbiology | 2190 | 73644 |
| microbial community | 2117 | 73815 |
| soil | 2074 | 68798 |
| bacteria (microorganisms) | 2029 | 56677 |
| biodegradation | 1863 | 51631 |
| metals, heavy | 1751 | 58137 |
| phytoremediation | 1739 | 49203 |
| soils | 1687 | 55272 |
| chromium | 1679 | 49020 |
| pH | 1617 | 53271 |
| copper | 1550 | 50679 |
| biotechnology | 1524 | 39017 |
| lead | 1489 | 49019 |
| zinc | 1406 | 47921 |
| biomass | 1325 | 42641 |
| genetics | 1321 | 41617 |
| soil microbiology | 1295 | 45335 |
| enzyme activity | 1261 | 42738 |
| arsenic | 1253 | 37189 |
| microbial activity | 1216 | 37123 |
| bioaccumulation | 1197 | 38282 |
| water pollutants, chemical | 1196 | 40156 |
| bacterial strain | 1188 | 38708 |
| toxicity | 1152 | 34752 |
| water pollutant | 1147 | 38784 |
| rna 16s | 1061 | 38235 |
| adsorption | 1044 | 31365 |
| contamination | 1040 | 31238 |
| unclassified drug | 1027 | 33732 |
| pollutant removal | 1006 | 30548 |
| microorganisms | 1002 | 26144 |
| iron | 945 | 32534 |
| plant growth | 931 | 35295 |
| wastewater treatment | 928 | 26115 |
| heavy metal removal | 920 | 29488 |
| nitrogen | 876 | 31347 |
| pollution | 874 | 24573 |

**Supplementary Table S7** Bias sources.

| **Bias domain** | **Low risk** | **Moderate risk** | **High risk** | **Main source of bias** |
| --- | --- | --- | --- | --- |
| Matrix bias | Real soils | Soil microcosms | Synthetic solutions | Overestimation in batch systems |
| Performance bias | Field / pot trials | Short-term assays | Single-time-point batch | Inflated removal rates |
| Analytical bias | ICP-MS / ICP-OES | AAS | Colorimetric only | Detection limits |
| Reporting bias | Full parameters | Partial | Missing pH/replicates | Limited reproducibility |
| Mechanistic bias | Genetic + spectroscopic | Spectroscopic only | Descriptive | Weak causal inference |

**References**

Ali, Q., Ayaz, M., Yu, C., Wang, Y., Gu, Q., Wu, H., et al. (2022). Cadmium tolerant microbial strains possess different mechanisms for cadmium biosorption and immobilization in rice seedlings. *Chemosphere* 303, 135206. doi: 10.1016/j.chemosphere.2022.135206

Arce-Inga, M., González-Pérez, A. R., Hernandez-Diaz, E., Chuquibala-Checan, B., Chavez-Jalk, A., Llanos-Gomez, K. J., et al. (2022). Bioremediation Potential of Native *Bacillus* sp. Strains as a Sustainable Strategy for Cadmium Accumulation of Theobroma cacao in Amazonas Region. *Microorganisms* 10, 2108. doi: 10.3390/microorganisms10112108

Bhandari, D., Sharma, P., and Dwivedi, V. (2024). Microbial bioremediation: unraveling the potential for cleaner environments through comparative analysis. *Bioremediation J.*, 1–9. doi: 10.1080/10889868.2024.2357136

Cai, Q., Zhao, W., Wang, J., Yang, G., Amils, R., Martínez, J. M., et al. (2025). A cooperation mechanism between *Bacillus thuringiensis* and *Citrobacter freundii* that enhances cadmium biomineralization. *J. Hazard. Mater.* 488, 137354. doi: 10.1016/j.jhazmat.2025.137354

Chen, Y., Fang, Z., Iddrisu, L., Ye, J., Pan, X., Liang, Y., et al. (2025). Integrated analyses of characterization and transcriptome reveal the adaptive response mechanism of *Bacillus cereus* FCHN 7–1 in cadmium adsorption. *J. Hazard. Mater.* 489, 137525. doi: 10.1016/j.jhazmat.2025.137525

Dahnoun, K., Djadouni, F., Essghaier, B., Naccache, C., Zitouna, N., Zehdi-Azouzi, S., et al. (2024). Characterization and bioremediation potential of heavy-metal resistant bacteria isolated from agricultural soil. *Turk. J. Agric. For.* 48, 607–617. doi: 10.55730/1300-011X.3205

Elahi, A., Rehman, A., Zajif Hussain, S., Zulfiqar, S., and Shakoori, A. R. (2022). Isolation and characterization of a highly effective bacterium *Bacillus cereus b-525k* for hexavalent chromium detoxification. *Saudi J. Biol. Sci.* 29, 2878–2885. doi: 10.1016/j.sjbs.2022.01.027

Firincă, C., Zamfir, L.-G., Constantin, M., Răut, I., Capră, L., Popa, D., et al. (2023). Microbial Removal of Heavy Metals from Contaminated Environments Using Metal-Resistant Indigenous Strains. *J. Xenobiotics* 14, 51–78. doi: 10.3390/jox14010004

Gao, Y., Zhang, X., Wang, L., Guan, E., Zhu, L., Wang, J., et al. (2024). Contribution of Cd passivating functional bacterium H27 to tobacco growth under Cd stress. *Chemosphere* 362, 142552. doi: 10.1016/j.chemosphere.2024.142552

Ghosh, P. K., Pramanik, K., Mahapatra, K., Mondal, S., Ghosh, S. K., Ghosh, A., et al. (2024). Plant growth-promoting *Bacillus cereus* MCC3402 facilitates rice seedling growth under arsenic-spiked soil. *Biocatal. Agric. Biotechnol.* 61, 103405. doi: 10.1016/j.bcab.2024.103405

Henagamage, A. P., Peries, C. M., and Seneviratne, G. (2022). Fungal-bacterial biofilm mediated heavy metal rhizo-remediation. *World J. Microbiol. Biotechnol.* 38, 85. doi: 10.1007/s11274-022-03267-8

Huang, X., Zhang, R., Xu, Y., and Zheng, J. (2024). Immobilization of Cd2+ in an aqueous environment using a two-step microbial-induced carbonate precipitation method. *J. Environ. Manage.* 351, 119868. doi: 10.1016/j.jenvman.2023.119868

Kashyap, S., Chandra, R., Kumar, B., and Verma, P. (2022). Biosorption efficiency of nickel by various endophytic bacterial strains for removal of nickel from electroplating industry effluents: an operational study. *Ecotoxicology* 31, 565–580. doi: 10.1007/s10646-021-02445-y

Kaur, M., Sidhu, N., and Reddy, M. S. (2024). Removal of cadmium through biomineralization using halophilic and ureolytic bacteria under saline conditions. *Int. Biodeterior. Biodegrad.* 191, 105805. doi: 10.1016/j.ibiod.2024.105805

Kaushal, P., and Pati, A. M. (2024). *Bacillus altitudinis* mediated lead bioremediation for enhanced growth of rice seedlings. *Curr. Microbiol.* 81, 410. doi: 10.1007/s00284-024-03934-z

Liang, B., Feng, Y., Ji, X., Li, C., Li, Q., Zeng, Z., et al. (2025). Isolation and characterization of cadmium-resistant *Bacillus cereus* strains from Cd-contaminated mining areas for potential bioremediation applications. *Front. Microbiol.* 16, 1550830. doi: 10.3389/fmicb.2025.1550830

Lin, H., Shi, J., Dong, Y., Li, B., and Yin, T. (2022). Construction of bifunctional bacterial community for co-contamination remediation: Pyrene biodegradation and cadmium biomineralization. *Chemosphere* 304, 135319. doi: 10.1016/j.chemosphere.2022.135319

Manikandan, S. K., and Nair, V. (2023). Developing a biocatalyst showcasing the synergistic effect of rice husk biochar and bacterial cells for the removal of heavy metals. *New J. Chem.* 47, 21199–21213. doi: 10.1039/D3NJ02889E

Mitra, S., Dey, J., Sarkar, S., and Banik, P. (2025). Halotolerant bacteria isolated from the soils of Indian mangrove ecosystem for metal removal and NPK enhancement. *Sci. Rep.* 15, 20804. doi: 10.1038/s41598-025-07839-0

Mondal, S., Mondal, T., Pal, P., Mitra, S., Ghosh, S. K., Soren, T., et al. (2025). Bioprotective mechanisms of *Enterobacter* sp. against arsenic, cadmium, and lead toxicity and its potential role in soil bioremediation. *J. Environ. Chem. Eng.* 13, 115432. doi: 10.1016/j.jece.2025.115432

Monga, A., Fulke, A. B., Gaud, A., Sharma, A., Ram, A., and Dasgupta, D. (2022). Isolation and Identification of Novel Chromium Tolerant Bacterial Strains From a Heavy Metal Polluted Urban Creek: An Assessment of Bioremediation Efficiency and Flocculant Production. *Thalass. Int. J. Mar. Sci.* 38, 1233–1244. doi: 10.1007/s41208-022-00458-w

Mtengai, K., Ramasamy, S., Msimuko, P., Mzula, A., and Mwega, E. D. (2022). Existence of a novel heavy metal–tolerant *Pseudomonas aeruginosa* strain Zambia SZK-17 Kabwe 1: the potential bioremediation agent in the heavy metal–contaminated area. *Environ. Monit. Assess.* 194, 887. doi: 10.1007/s10661-022-10565-z

Mungla, G., Facknath, S., and Lalljee, B. (2022). Assessing the Potential of Mechanical Aeration Combined with Bioremediation Process in Soils and Coastal Sediments Impacted by Heavy Metals. *AIMS Environ. Sci.* 9, 692–707. doi: 10.3934/environsci.2022039

Peng, C., Zhao, X., Ji, X., Wu, J., Liang, W., Song, H., et al. (2023). Mixed bacteria passivation for the remediation of arsenic, lead, and cadmium: Medium optimization and mechanisms. *Process Saf. Environ. Prot.* 170, 720–727. doi: 10.1016/j.psep.2022.12.037

Sahu, S., Rajbonshi, M. P., Gujre, N., Gupta, M. K., Shelke, R. G., Ghose, A., et al. (2022). Bacterial strains found in the soils of a municipal solid waste dumping site facilitated phosphate solubilization along with cadmium remediation. *Chemosphere* 287, 132320. doi: 10.1016/j.chemosphere.2021.132320

Satyapal, G. K., Haque, R., and Kumar, N. (2024). Gamma irradiation in modulating arsenic bioremediation potential of *Pseudomonas* sp. AK1 and AK9. *Int. J. Radiat. Biol.* 100, 934–939. doi: 10.1080/09553002.2024.2345137

Shaaban Emara, N., Elwakil, B. H., Zakaria, M., and Olama, Z. A. (2023). Mechanistic action of lead removal by the native isolate *Bacillus amyloliquefaciens* ON261680.1. *Arab. J. Chem.* 16, 104962. doi: 10.1016/j.arabjc.2023.104962

Shan, B., Hao, R., Zhang, J., Li, J., Ye, Y., Xu, H., et al. (2024). Bioremediation Potential of Cr(VI) by *Lysinibacillus cavernae* CR-2 Isolated from Chromite-Polluted Soil: A Promising Approach for Cr(VI) Detoxification. *Geomicrobiol. J.* 41, 459–473. doi: 10.1080/01490451.2023.2246035

Sizentsov, A. N., Galaktionova, L. V., Davydova, O. K., and Nikiyan, H. N. (2025). In vitro assessment of the biosorption potential of some representatives of the genus bacillus during interaction with lead cations. *Appl. Ecol. Environ. Res.* 23, 4335–4349. doi: 10.15666/aeer/2303_43354349

Soto-Ramírez, R., Vlatten, N., Ruz, F., Tavernini, L., and Lobos, M.-G. (2024). Engineering the cell wall reactive groups of Plant Growth Promoting Rhizobacteria by culture strategy for heavy metal removal. *J. Biotechnol.* 394, 125–134. doi: 10.1016/j.jbiotec.2024.08.015

Su, Y., Sun, S., Liu, Q., Zhao, C., Li, L., Chen, S., et al. (2022). Characterization of the simultaneous degradation of pyrene and removal of Cr(VI) by a bacteria consortium YH. *Sci. Total Environ.* 853, 158388. doi: 10.1016/j.scitotenv.2022.158388

Sundarraj, S., Sudarmani, D. N. P., Samuel, P., and Sevarkodiyone, S. P. (2023). Bioremediation of hexavalent chromium by transformation of *Escherichia coli* DH5α with chromate reductase (ChrR) genes of *Pseudomonas putida* isolated from tannery effluent. *J. Appl. Microbiol.* 134, lxac019. doi: 10.1093/jambio/lxac019

Surabhi, A., Sundaramanickam, A., Barathan, V., and Praveena, S. M. (2025). Multi-metal Tolerance Efficiency and Bioremediation Capabilities of *Bacillus aerius* from Mangrove Rhizosphere. *Geomicrobiol. J.* 42, 737–744. doi: 10.1080/01490451.2025.2523818

Wang, X., Xu, W., Lu, Y., Cao, L., Tian, M., Shang, Y., et al. (2025). Lead biosorption by *Bacillus tequilensis*: characterization of resistance mechanisms and its potential for remediation of contaminated soil. *J. Sci. Food Agric.* 105, 7095–7102. doi: 10.1002/jsfa.14408

Yan, Z., Li, Y., Peng, S., Wei, L., Zhang, B., Deng, X., et al. (2024). Cadmium biosorption and mechanism investigation using two cadmium-tolerant microorganisms isolated from rhizosphere soil of rice. *J. Hazard. Mater.* 470, 134134. doi: 10.1016/j.jhazmat.2024.134134

Yasmin, R., Zafar, M. S., Tahir, I. M., Asif, R., Asghar, S., and Raza, S. K. (2022). Biosorptive Potential of *Pseudomonas* species RY12 Toward Zinc Heavy Metal in Agriculture Soil Irrigated with Contaminated Waste Water. *Dose-Response* 20, 15593258221117352. doi: 10.1177/15593258221117352

Ye, Y., Hao, R., Shan, B., Zhang, J., Li, J., and Lu, A. (2024). Mechanism of Cr(VI) removal by efficient Cr(VI)-resistant *Bacillus mobilis* CR3. *World J. Microbiol. Biotechnol.* 40, 21. doi: 10.1007/s11274-023-03816-9

Zhang, F., Deng, Y., Peng, R., Jiang, H., and Bai, L. (2024a). Bioremediation of paddy soil with amphitropic mixture markedly attenuates rice cadmium: Effect of soil cadmium removal and Fe/S-cycling bacteria in rhizosphere. *Sci. Total Environ.* 915, 169876. doi: 10.1016/j.scitotenv.2024.169876

Zhang, L., Hu, Y., Chen, Y., Qi, D., Cai, B., Zhao, Y., et al. (2023). Cadmium-tolerant *Bacillus cereus* 2–7 alleviates the phytotoxicity of cadmium exposure in banana plantlets. *Sci. Total Environ.* 903, 166645. doi: 10.1016/j.scitotenv.2023.166645

Zhang, L., Wang, W., Yue, C., and Si, Y. (2024b). Biogenic calcium improved Cd2+ and Pb2+ immobilization in soil using the ureolytic bacteria *Bacillus pasteurii*. *Sci. Total Environ.* 921, 171060. doi: 10.1016/j.scitotenv.2024.171060

Zuo, W., Song, B., Shi, Y., Zupanic, A., Guo, S., Huang, H., et al. (2022). Using *Bacillus thuringiensis* HM-311 hydroxyapatite biochar beads to remediate Pb and Cd contaminated farmland soil. *Chemosphere* 307, 135797. doi: 10.1016/j.chemosphere.2022.135797
